# Genetic associations in polypoidal choroidal vasculopathy: A systematic review and meta-analysis

**Published:** 2012-04-04

**Authors:** Haoyu Chen, Ke Liu, Li Jia Chen, Ping Hou, Weiqi Chen, Chi Pui Pang

**Affiliations:** 1 Joint Shantou International Eye Center, Shantou University & the Chinese University of Hong Kong, Shantou, China; 2 Department of Ophthalmology and Visual Sciences, the Chinese University of Hong Kong, Hong Kong, China

## Abstract

**Purpose:**

To investigate the genetic associations of polypoidal choroidal vasculopathy (PCV), the genetic difference between PCV and age-related macular degeneration (AMD), and the genotype-phenotype correlation of PCV.

**Methods:**

A systematic review and meta-analysis were performed. Published articles about genetic associations of PCV identified from a literature search were reviewed. The following data from individual studies were extracted and analyzed: 1) comparison of genetic polymorphisms between PCV and controls; 2) comparison of genetic polymorphisms between PCV and AMD; and 3) comparison of phenotypes between different genotype groups.

**Results:**

A total of 33 articles fulfilled the inclusion criteria. With meta-analyses, variants in four genes were found to be significantly associated with PCV: *LOC387715* rs10490924 (n=9, allelic odds ratio [OR]=2.27, p<0.00001), *HTRA1* rs11200638 (n=4, OR=2.72, p<0.00001), *CFH* rs1061170 (n=4, OR=1.72, p<0.00001), *CFH* rs800292 (n=5, OR=2.10, p<0.00001), and *C2* rs547154 (n=3, OR=0.56, p=0.01). *LOC387715* rs10490924 was the only variant showing a significant difference between PCV and wet AMD (n=5, OR=0.66, p<0.00001). The risk genotypes of rs10490924 were associated with larger lesion size, greater chance of vitreous hemorrhage, and worse therapeutic response in PCV.

**Conclusions:**

*LOC387715* rs10490924 was associated with PCV and its clinical manifestations, and showed a discrepant distribution between PCV and AMD. Variants in *HTRA1*, *CFH*, and *C2* were also associated with PCV.

## Introduction

Polypoidal choroidal vasculopathy (PCV) is a sight-threatening disease in older adults. Clinically, it shares several common manifestations with wet age-related macular degeneration (AMD) such as subretinal exudation and hemorrhage involving the macular region. PCV was first identified as distinct from wet AMD in 1990 by Yannuzzi et al. [1], who reported a series of patients with peculiar polypoidal, subretinal vascular lesions associated with serous and hemorrhagic detachment of the retinal pigment epithelium, and named the clinical subtype PCV. Later, researchers reported that under indocyanine green angiography (ICGA), PCV demonstrated the distinct characteristic of a branching network of vessels and dilation at the ends of the vascular network in the inner choroid [2], thus making ICGA a standard investigation for diagnosing PCV.

The etiology of PCV remains largely unknown. It is likely a multifactorial disease sharing some mechanisms with AMD. To date, it is well recognized that genetic factors play an important role in the pathogenesis of AMD. In 2005, a coding single nucleotide polymorphism (SNP) rs1061170 (Y402H) at the Complement factor H (*CFH*) gene was found to be strongly associated with AMD in a genome-wide association study (GWAS) [3-7] in Caucasians. Subsequently, the SNP rs10490924 at *LOC387715* (also known as *ARMS2*) and rs11200638 at *HTRA1* were found to be associated with AMD in Caucasian and Asian populations [8-11]. Later, other genes or loci were also discovered as risk or protective factors for AMD, such as Complement factor B (*CFB)* [12], Complement factor C2 [12], Complement factor C3 [13], *SERPING1* [14], and so on. The phenotypic similarities between AMD and PCV lead to the hypothesis that genes involved in AMD may also play a role in PCV. Therefore, investigating AMD genes involved in PCV may easily reveal candidate genes for PCV [15,16], providing insights into the pathogenesis of PCV; in addition, differentiating the genetic profiles between PCV and AMD may provide clues to whether PCV is a subtype of wet AMD or a distinct disease [17], shedding some light on the pathogenesis of the respective phenotypes. Furthermore, genotype-phenotype correlation analysis, especially genetic predictors of therapy, may provide guidelines for better management of patients with AMD or PCV [18]. Thus far, however, findings on the genetic profiles of PCV compared to AMD remain controversial among different reports [17,19,20].

Here, in an attempt to give the overall effects and solve the controversies, we report a systematic review and meta-analysis summarizing all published results of genetic associations in PCV. This study 1) investigated which genetic variants are significantly associated with PCV and their effect sizes, 2) examined whether there are differences between the genetic risks of PCV and AMD, and 3) summarized the results of genotype-phenotype correlations in PCV.

## Methods

### Literature search

A systematic literature search using the databases PubMed, Embase, Web of Science, and the Chinese Biomedical Database was conducted on October 30, 2011, to identify all published studies on the association of genetic polymorphisms with PCV and/or its phenotypes. The search strategy (polypoidal choroidal vasculopathy OR PCV) AND (gene OR genetic OR polymorphism OR variant OR DNA or SNP) in All Fields was used. We did not use the option of language limitation on PubMed, Embase, or Web of Science.

### Review process

Two reviewers (H.Y.C. and K.L.) independently reviewed the retrieved records. The following inclusion criteria were applied during the review process: 1) association study of genetic variants with PCV and/or its phenotypes; 2) raw data of allele or genotype frequencies or counts available; 3) the type of article an original research study, not a review, case report, or editorial comment. For studies published by the same group on the same gene and markers, only the most recent article or the article with the largest sample size was included for analysis. Independent review and resolution by a third reviewer (L.J.C.) was sought if the two reviewers disagreed.

### Data extraction

The following data were extracted: author, year of publication, ethnicity of study subjects, whether the Hardy–Weinberg equilibrium (HWE) was examined in controls, the numbers and demographic characteristics of the patients and controls, and the allele and genotype counts or frequencies of each SNP in the patients and controls. The allele counts were calculated from the genotype counts when needed. We also calculated the allele or genotype counts from the frequencies, rounding to the closest integer, for studies [21,22] in which the genotype counts were not given. For the analysis of the genotype-phenotype correlation, the following phenotype information were extracted at each genotype group: the gender and bilaterality counts, the case number, the mean and standard deviation of age of onset, the best-corrected visual acuity (BCVA), greatest linear diameter (GLD) of lesion on fluorescence fundus angiography (FFA) and ICGA, and BCVA at 12 months after photodynamic therapy or combined therapy. For the study [23] in which the standard deviation was not given, we obtained it by communicating with the corresponding author. The data extraction and data input processes were performed by two reviewers (H.Y.C. and K.L.) independently. Further independent review and resolution by a third reviewer (L.J.C.) was sought if the two reviewers disagreed.

### Data analysis

To investigate the associations of gene variants with PCV, the allele and genotype frequencies of the SNPs were compared between PCV and normal controls. Six genetic models were used in the association analysis: allele, homozygote, heterozygote, dominant, recessive, and additive. To investigate whether PCV and wet AMD have different genetic risks, the allele frequencies of the SNPs were compared between the patients with PCV and wet AMD in the studies that included both disorders. To investigate the genotype-phenotype correlation, the phenotype characters were compared between patients carrying one or two risk alleles and patients without the risk allele. The results of individual studies were pooled using the software Review Manager (RevMan, version 5.1.4, The Cochrane Collaboration, Copenhagen, Denmark). In all of the meta-analyses, the odds ratios (ORs) or mean differences (MDs) and 95% confidence interval (CIs) were estimated with the fixed or random model according to the heterogeneity test. When the heterogeneity test α was <0.1, a random model was applied; otherwise, a fixed model was applied. Egger’s test was used to evaluate possible publication bias in the meta-analysis with the number of included studies >2.

## Results

**Figure 1:**
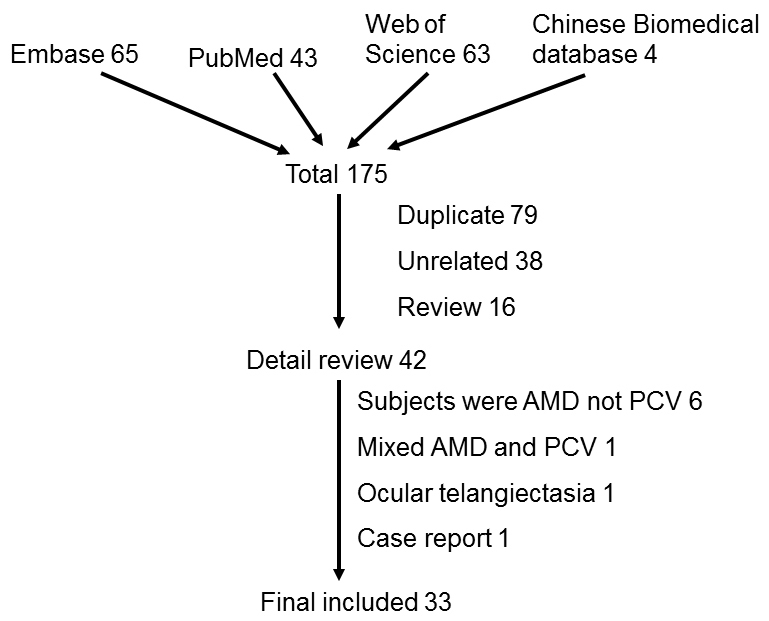
Flow diagram of literature screening. Flow diagram depicted the screening process of retrieved articles, including the number and reason of exclusion.

A total of 175 articles were identified from literature search, including 43 from PubMed, 63 from Embase, 65 from Web of Science, and four from the Chinese Biomedical Literature Database. Among them, 79 were excluded because of duplication, 38 were excluded because of unrelated topics, and 16 were excluded because of the publication type, such as a review. The full text of the remaining 42 records was retrieved and reviewed. Nine articles were excluded after the full text was reviewed (Figure 1). Finally, 33 articles were included for the meta-analysis. All the studies were case-control studies, and none was family based. The characteristics of these articles are listed in Table 1.

**Table 1:** Characteristics of the included studies

**Author**	**Year**	**Ethnicity**	**HWE**	**PCV**	**AMD**	**Control**	**Gene(s)/locus investigated**	**Ref.**
Gotoh	2004	Japanese	yes	58	85	82	APOE	[15]
Gotoh	2008	Japanese	no	204	116	-	HTRA1, CFH	[35]
Gotoh	2009	Japanese	yes	55	56	77	LOC387715	[24]
Gotoh	2010	Japanese	yes	181	84	276	LOC387715, HTRA1	[25]
Yamashiro	2011	Japanese	no	518	408	336	Elastin	[20]
Yamashiro	2011	Japanese	no	154	216	142	Elastin	[20]
Nakanishi	2010	Japanese	yes	375	-	847	LOC387715, CFH	[26]
Hayashi	2010	Japanese	yes	518	408	1351	LOC387715, CFH	[27]
Nakata	2011	Japanese	yes	167	-	-	PEDF, SERPINF1	[49]
Nakata	2011	Japanese	yes	510	401	336	SERPING1	[37]
Tsujikawa	2011	Japanese	no	88	-	-	LOC387715	[45]
Kondo	2007	Japanese	yes	76	73	94	LOC387715, HTRA1	[16]
Kondo	2008	Japanese	yes	103	78	104	Elastin	[17]
Kondo	2009	Japanese	yes	130	-	173	CFH	[21]
Kondo	2009	Japanese	yes	136	-	183	CFB, C2, RDBP, SKIV2L	[21]
Kondo	2009	Japanese	yes	140	116	189	SOD2	[40]
Bessho	2009	Japanese	yes	140	116	189	PEDF	[38]
Bessho	2011	Japanese	no	119	68	-	LOC387715	[34]
Sakurada	2008	Japanese	yes	109	-	85	LOC387715	[30]
Sakurada	2009	Japanese	yes	92	-	-	LOC387715	[44]
Sakurada	2010	Japanese	yes	71	-	-	LOC387715	[18]
Sakurada	2011	Japanese	yes	226	-	-	LOC387715, CFH	[23]
Goto	2009	Japanese	yes	100	100	190	LOC387715, CFH, C3	[29]
Fuse	2011	Japanese	yes	60	50	138	LOC387715, LOXL1	[28]
Tanaka	2011	Japanese	yes	287	-	277	LOC387715, CFH	[31]
Park	2011	Korea	yes	103	-	112	LOC387715, HTRA1	[33]
Park	2011	Korea	yes	51	-		LOC387715, HTRA1	[43]
Li	2010	Chinese	yes	118	-	115	SERPING1	[36]
Zhang	2011	Chinese	yes	177	131	182	9p21	[42]
Wu	2011	Chinese	yes	177	131	182	PEDF	[39]
Sng	2011	Chinese	yes	120	126	274	Toll-like receptor 3	[41]
Lee	2008	Chinese	yes	72	-	93	LOC387715, CFH, CFB, C2	[32]
Lima	2011	Caucasian	no	56	368	368	Elastin	[19]
Lima	2010	Caucasian	no	55	368	368	LOC387715, CFH, CFB, C2	[22]

HWE: Hardy–Weinberg equilibrium; PCV: polypoidal choroidal vasculopathy; AMD: Age-related macular degeneration. Ref: Reference

**Figure 2:**
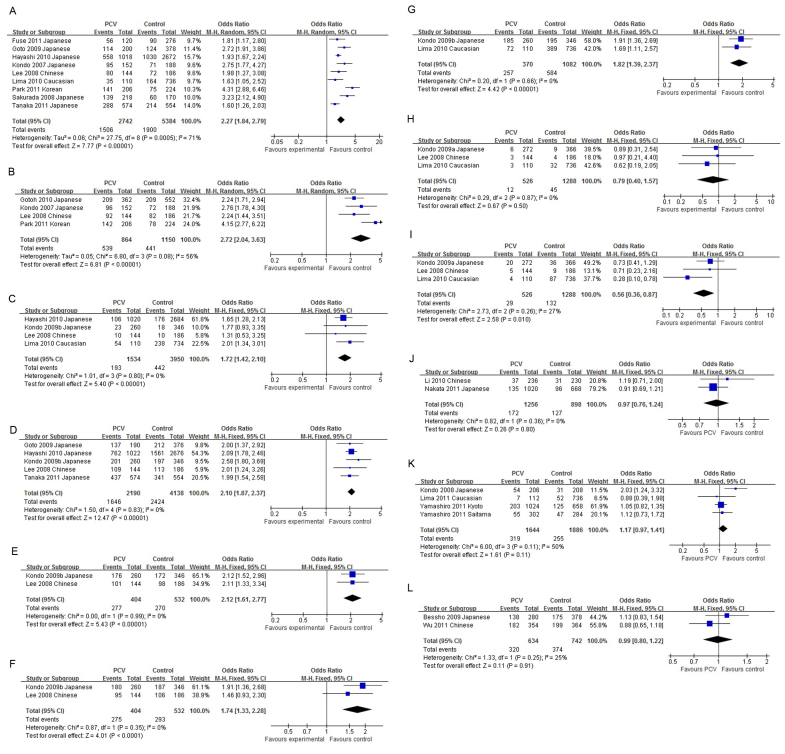
The forest plots of meta-analysis compared the allelic frequencies between polypoidal choroidal vasculopathy and control. Squares indicate the study-specific odds ratio (OR). The size of the box is proportional to the weight of the study. Horizontal lines indicate 95% confidence interval (CI). A diamond indicates the summary OR with its corresponding 95% CI. **A**: *LOC387715* rs10490924; **B**: *HTRA1* rs11200638; **C**: Complement factor H (*CFH*) rs1061170; **D**: *CFH* rs800292; E: *CFH* rs3753394; F: *CFH* rs1329428; **G**: *CFH* rs1410996; **H**: *CFB* rs415667; **I**: *C2* rs547154; **J**: *SERPING1* rs2511989; **K**: Elastin rs2301995; **L**: *PEDF* rs1136287.

**Figure 3:**
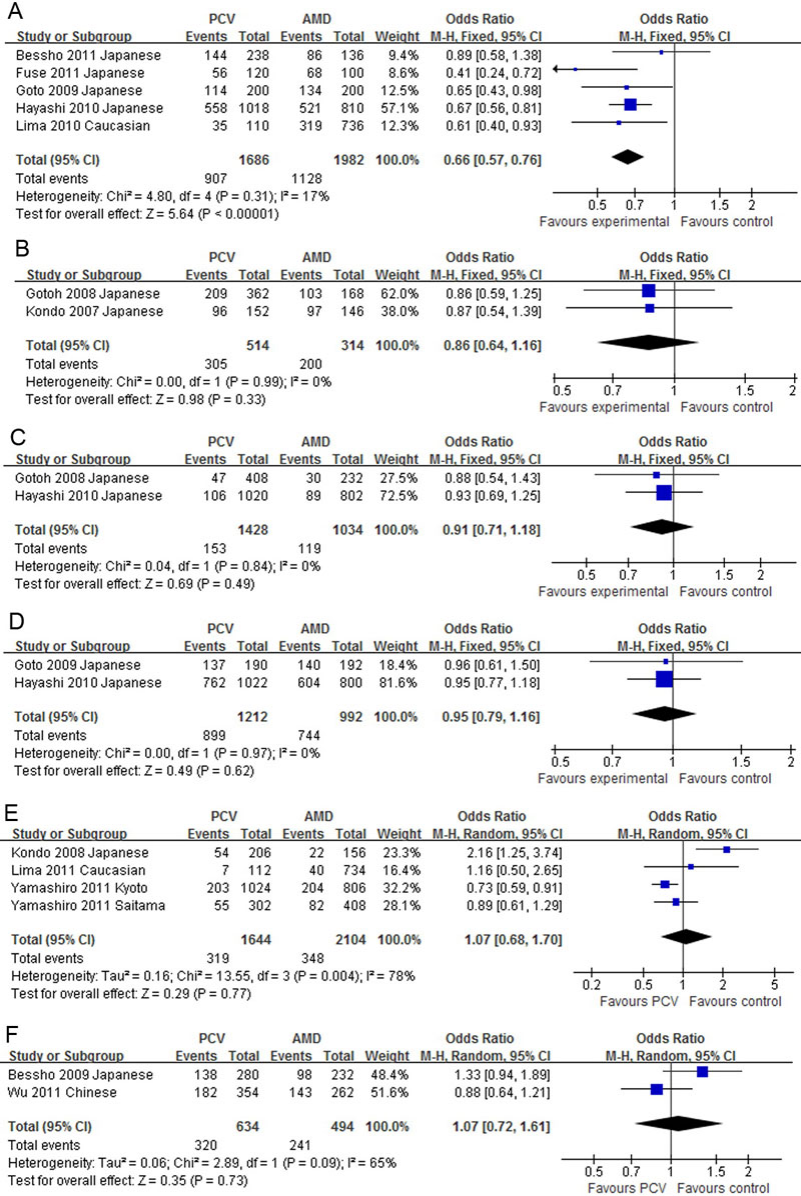
The forest plots of meta-analysis compared the allelic frequencies between polypoidal choroidal vasculopathy and age-related macular degeneration. Squares indicate the study-specific odds ratio (OR). The size of the box is proportional to the weight of the study. Horizontal lines indicate 95% confidence interval (CI). A diamond indicates the summary OR with its corresponding 95% CI. **A**: *LOC387715* rs10490924; **B**: *HTRA1* rs11200638; **C**: Complement factor H (*CFH*) rs1061170; **D**: *CFH* rs800292; **E**: Elastin rs2301995; **F**: *PEDF* rs1136287.

All reported genetic associations in PCV are summarized in Table 2. The SNP rs10490924 at *LOC387715* was the most investigated SNP in PCV. Four articles [24-27] were from the same study group; only the most recent was used [27]. Nine studies were included in the meta-analysis [16,22,27-33]. The minor allele, T, was more frequent in PCV than in the controls in all articles. The allelic OR in an individual study ranged from 1.63 to 4.31, with a pooled OR of 2.27 (95% CI: 1.84–2.79, p<0.00001, Figure 2A). No significant publication bias was detected (Egger’s test p=0.202). The pooled ORs were 4.90, 1.74, 2.44, 3.26, and 1.65 for the homozygote, heterozygote, dominant, recessive, and additive models, respectively, with all p<0.0001 (Appendix 1**)**. The allele frequencies of rs10490924 in PCV and AMD were reported in five studies [22,27-29,34]. The T allele frequency was lower in PCV compared to AMD in all reports, with a pooled OR of 0.66 (95% CI: 0.57–0.76, p<0.00001, Figure 3A). No significant publication bias was detected (Egger’s test p=0.627).

**Table 2:** Summary of allelic odds ratios of gene variants in polypoidal choroidal vasculopathy

**Gene/locus**	**SNP**	**Allele**	**PCV versus control OR (95% CI)**	**Case**	**Control**	**N**	**Ref**	**PCV versus AMD OR (95% CI)**	**N**	**Ref**
LOC387715	rs10490924	T	2.27 (1.84–2.79)	2742	5383	9	Figure 2A	0.66 (0.57–0.76)	5	Figure 3A
HTRA1	rs11200638	A	2.72 (2.04–3.63)	864	1150	4	Figure 2B	0.86 (0.64–1.16)	2	Figure 3B
CFH	rs1061170	C	1.72 (1.42–2.10)	1534	3950	4	Figure 2C	0.91 (0.71–1.18)	2	Figure 3C
CFH	rs800292	G	2.10 (1.87–2.37)	2190	4138	5	Figure 2D	0.95 (0.79–1.16)	2	Figure 3D
CFH	rs3753394	T	2.12 (1.61–2.77)	404	532	2	Figure 2E	NA		
CFH	rs1329428	T	1.74 (1.33–2.28)	404	532	2	Figure 2F	NA		
CFH	rs1410996	C	1.82 (1.39–2.37)	370	1082	2	Figure 2G	0.71 (0.47–1.06)	1	[22]
CFB	rs415667	A	0.79 (0.40–1.57)	526	1288	3	Figure 2H	1.35 (0.38–4.73)	1	[22]
C2	rs547154	T	0.56 (0.36–0.87)	526	1288	3	Figure 2I	0.73 (0.26–2.10)	1	[22]
SERPING1	rs2511989	A	0.97 (0.76–1.24)	1256	898	2	Figure 2J	0.91 (0.70–1.20)	1	[37]
Elastin	rs2301995	G	1.17 (0.97–1.41)	1644	1886	4	Figure 2K	1.07 (0.68–1.70)	4	Figure 3E
PEDF	rs1136287	T	0.99 (0.80–1.22)	634	742	2	Figure 2L	1.07 (0.72–1.61)	2	Figure 3F
SOD2	rs4880	C	0.81 (0.52–1.26)	140	189	1	[40]	1.11 (0.66–1.88)	1	[40]
TLR3	rs3775291	T	1.27 (0.93–1.73)	120	274	1	[41]	1.16 (0.81–1.66)	1	[41]
9p21	rs10757278	A	1.44 (1.08–1.94)	177	182	1	[42]	1.12 (0.81–1.54)	1	[42]
RDBP	rs3880457	C	0.31 (0.13–0.71)	136	183	1	[21]	NA		
SKIV2L	rs2075702	C	0.31 (0.13–0.71)	136	183	1	[21]	NA		
C3	rs2241394	C	3.47 (1.48–8.38)	100	190	1	[29]	NA		

SNP: single nucleotide polymorphism; PCV: polypoidal choroidal vasculopathy; AMD: age-related macular degeneration; OR: odds ratio; CI: confidence interval; NA: not available. N: number of study cohorts. Ref: reference.

The SNP rs11200638 located at the promoter of *HTRA1* was investigated in five studies [16,25,32,33,35]. Two articles [25,35] were from the same study group, and the earlier one [35] was excluded. The A allele was more prevalent in PCV than in the controls in all studies, with individual allelic ORs ranging from 2.24 to 4.15. The pooled allelic OR was 2.72 (95% CI: 2.04–3.63, p<0.00001, Figure 2B). No significant publication bias was detected (Egger’s test p=0.539). The pooled ORs were 6.43, 1.75, 2.95, 4.50, and 2.08 in the homozygote, heterozygote, dominant, recessive, and additive models, respectively (all p<0.001, Appendix 2). The distributions of rs11200638 in PCV and AMD were reported in two studies [16,25]. Although the frequencies of the A allele in PCV were lower than in AMD in both studies, neither the individual ORs nor the pooled OR (0.86, 95% CI: 0.64–1.16, p=0.33, Figure 3B) was statistically significant.

The association of *CFH* rs1061170 (Y402H) with PCV was investigated in five studies [21,22,27,32,35], among which one [35] was excluded because the authors were from the same group as those in a later article [27]. The C allele was more frequent in PCV than in the controls. The OR in an individual study was statistically significant in two studies [22,27] but not in the other two [21,32]. The pooled allelic OR was 1.72 (95% CI: 1.42–2.10, p<0.000001, Figure 2C). No significant publication bias was detected (Egger’s test p=0.912). The pooled ORs were statistically significant in the heterozygote (1.72), dominant (1.71), and additive (1.57) models, but not for the homozygote (1.48) or recessive (1.37) model (Appendix 3). There was no significant difference in the allele frequencies of rs1061170 between PCV and AMD, with a pooled OR of 0.91 (95% CI: 0.71–1.18, p=0.49, Figure 3C). The association of *CFH* rs800292 (I62V) with PCV was investigated in five studies [21,27,29,31,32]. The G allele was more frequent in PCV than in the controls, with a pooled allelic OR of 2.10 (95% CI: 1.87–2.37, p<0.00001, Figure 2D). No significant publication bias was detected (Egger’s test, p=0.780). The pooled ORs were 4.06, 1.92, 2.81, 2.42, and 1.67 in the homozygote, heterozygote, dominant, recessive, and additive models, respectively, with all p<0.00001 (Appendix 4). The allelic distributions of rs800292 in PCV and AMD were reported in two studies [27,29], but neither the individual studies nor the pooled analysis (OR=0.95, 95% CI: 0.79–1.16, p=0.62, Figure 3D) found a significant difference between PCV and AMD. The *CFH* rs3753394 was reported in two studies [21,32] and the pooled OR was 2.12 for the T allele (95% CI: 1.61–2.77, p<0.00001, Figure 2E). The *CFH* rs1329428 was reported in two studies [21,32] and the pooled OR was 1.74 for the C allele (95% CI: 1.33–2.28, p<0.0001, Figure 2F). The *CFH* rs1410996 was reported in two studies [21,22], and the pooled OR was 1.82 for the C allele (95% CI: 1.39–2.37, p<0.0001, Figure 2G).

At the *CFB-C2* locus, the association of *CFB* rs415667 with PCV was investigated in three studies [21,22,32]. Neither the individual OR nor the pooled OR (0.79, 95% CI: 0.40–1.57, p=0.50, Figure 2H) was statistically significant. No significant publication bias was detected (Egger’s test p=0.907). Only one study compared the allele frequency of rs415667 between PCV and wet AMD, and the OR was 1.35 (p=0.50) [22]. The association of *C2* rs547154 with PCV was investigated in three studies [21,22,32]. The individual ORs for the T allele ranged from 0.28 to 0.73, and only one was statistically significant [22]. The pooled OR was 0.56 (95% CI 0.36–0.87, p=0.01, Figure 2I). No significant publication bias was detected (Egger’s test p=0.601). Only one study compared the allele frequency of rs547154 between PCV and wet AMD, and the OR was 0.74 (p=0.82) [22].

Regarding the other genes, the SNP rs2511989 at the *SERPING1* gene was reported in two studies [36,37], but none of the original studies or the pooled analysis (OR=0.97, 95% CI: 0.76–1.24, p=0.80, Figure 2J) showed a statistically significant association with PCV. A significant association between rs2301995 in elastin and PCV was reported in one study cohort [17], but not in the other three cohorts [19,20]. The pooled OR was 1.17 (95% CI: 0.97–1.41, p=0.11, Figure 2K). No significant publication bias was detected (Egger’s test p=0. 0.721). A significant difference in the distributions of rs2301995 between PCV and AMD was shown in one cohort [17] but not in the other three [19,20], and the pooled OR was 1.07 (95% CI: 0.68–1.70, p=0.77, Figure 3E, Egger’s test p=0.233). Association of *PEDF* rs1136287 with PCV was reported in two studies [38,39], but no individual studies or the pooled analysis showed a significant association (Figure 2L). The allelic distributions of rs1136287 in PCV and AMD were not significantly different (Figure 3F). The associations of SNPs at *SOD2* [40], *PEDF* [38], *TLR3* [41], *9p21* [42], *RDBP* [21], *SKIV2L* [21], and *C3* [29] with PCV were reported in one article (Table 2). No meta-analysis was performed on these genes/loci.

**Figure 4:**
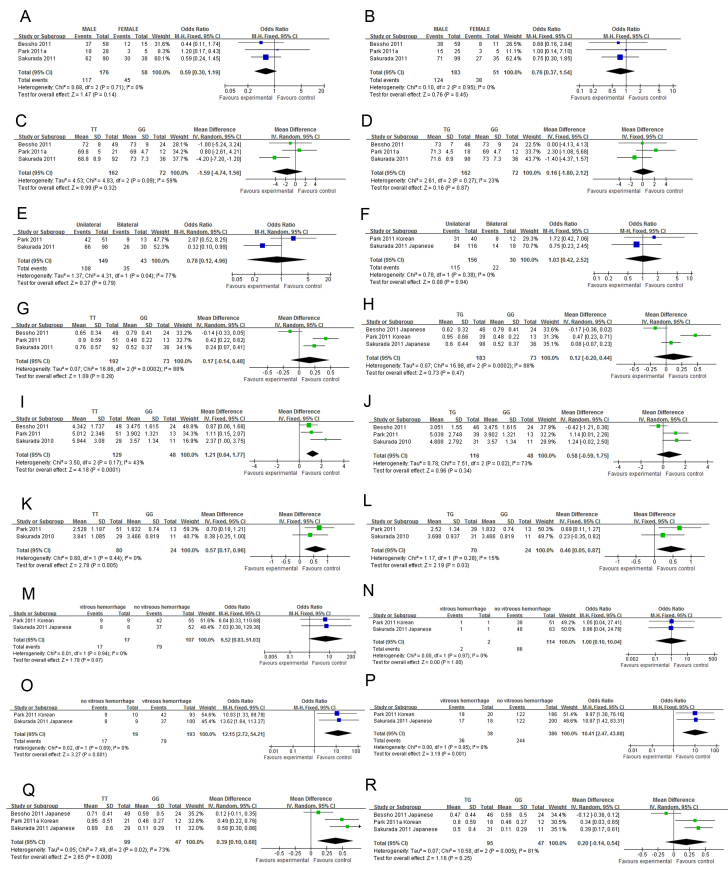
The forest plots of meta-analysis compared the phenotypes of polypoidal choroidal vasculopathy between different genotypes of *LOC387715* rs10490924. Blue squares indicate the study-specific odds ratio (OR). Green squares indicate the study-specific mean difference (MD). The size of the box is proportional to the weight of the study. Horizontal lines indicate 95% confidence interval (CI). A diamond indicates the summary OR (blue) or MD (green) with its corresponding 95% CI. **A**, **C**, **E**, **G**, **I**, **K**, **M** and **Q**: comparison between TT and GG; **B**, **D**, **F**, **H**, **J**, **L**, **N** and **R**: comparison between TG and GG. **O**: comparison between TT and TG + GG; **P**: comparison between T and G allele. **A** and **B**: Gender distribution; **C** and **D**: Age of onset; **E** and **F**: Bilaterality; **G** and **H**: Best-corrected visual acuity; **I** and **J**: Greatest linear diameter on fundus fluorescence angiography; **K** and **L**: Greatest linear diameter on indocyanine green angiography; **M**-**P**: Vitreous hemorrhage; **Q** and **R**: Best-corrected visual acuity at 12 months after photodynamic therapy or combined therapy.

**Figure 5:**
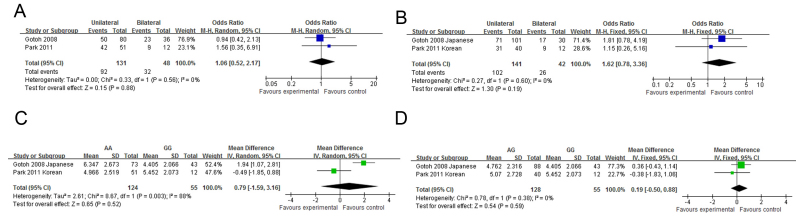
The forest plots of meta-analysis compared the phenotypes of polypoidal choroidal vasculopathy between different genotypes of *HTRA1* rs11200638. Blue squares indicate the study-specific odds ratio (OR). Green squares indicate the study-specific mean difference (MD). The size of the box is proportional to the weight of the study. Horizontal lines indicate 95% confidence interval (CI). A diamond indicates the summary OR (blue) or MD (green) with its corresponding 95% CI. **A** and **C**: Comparison between TT and GG; **B** and **D**: Comparison between TG and GG. **A** and **B**: Bilaterality; **C** and **D**: Greatest linear diameter on fundus fluorescence angiography.

Genotype-phenotype correlations of *LOC387715* rs10490924 were investigated in nine articles [18,23,30,31,33,34,43-45]. Four articles [18,23,30,44] were written by Sakurada’s group and another two [33,43] by Park’s group. Only the latest or largest data were included in the meta-analysis. Correlation of gender with rs10490924 was investigated in three studies [23,34,43]. Neither the original studies nor the meta-analysis showed a statistically significant difference in gender between different genotype groups (Figure 4A,B). One study [23] reported the mean age of onset in the TT genotype group was younger than in the GG genotype group. However, two other studies [34,43] and the pooled analysis showed a lack of significant difference in age of onset between different genotype groups (Figure 4C,D). A study reported that patients with the TT genotype of rs10490924 were more likely to have bilateral involvement compared to patients with the GG genotype [23]. However, a lack of significant correlation was reported in another study [33], and revealed by our meta-analysis (Figure 4E). Two studies reported that patients with the risk genotypes TT and TG had worse BCVA compared to those with the GG genotype [23,33]. However, in another study the result was reversed, although the difference was not statistically significant [34]. The meta-analysis did not support a significant difference in BCVA between the genotype groups (Figure 4G,H). Studies have reported that the FFA GLD in patients with the TT genotype was larger compared to those with the GG genotype in three studies [18,33,34]. Pooled analyses showed the mean difference was 1.21 mm (TT versus GG, 95% CI: 0.64–1.77, p<0.0001, Figure 4I), while the mean difference in FFA GLD between the TG and GG genotype groups was not statistically significant (Figure 4J). There was also a statistically significant difference in the ICGA GLD between the TT and GG genotype groups (pooled MD=0.57 mm, 95% CI: 0.17–0.96 mm, p=0.005, Figure 4K), as well as between the TG and GG genotype groups (pooled MD=0.46 mm, 95% CI: 0.05–0.87, p=0.03, Figure 4L). A study also reported that the T allele or TT genotype was associated with larger PCV (lesion size greater than 1 disc diameter) [45]. However, no mean or standard deviation for each genotype was reported in the article; therefore, it was not included in the meta-analysis. Association of rs10490924 with the risk of vitreous hemorrhage was reported in two studies [30,33]. The pooled OR was 6.52 (95% CI: 0.83–51.03, p=0.07, Figure 4M) and 1.00 (95% CI: 0.10–10.04, p=1.00, Figure 4N) for homozygote and heterozygote, respectively. Since the heterozygote OR was 1, we analyzed it using the recessive and allelic models, and the pooled ORs were 12.15 (95% CI: 2.72–54.21, p=0.001, Figure 4O) and 10.41 (95% CI: 2.47–43.88, p=0.001 Figure 4P), respectively. The BCVA 12 months after PDT or combined therapy was better in the GG genotype group than in the TT genotype group; the mean difference was 0.39 LogMAR (95% CI 0.10–0.68, p=0.008, Figure 4Q). The mean difference between the TG and GG genotype groups was 0.20 LogMAR lines but was not statistically significant (p=0.20, Figure 4R). The associations of bilaterality and lesion size of PCV with *HTRA1* rs11200638 were also investigated [33,35]. However, neither the original studies nor the pooled analysis showed a significant correlation (Figure 5).

## Discussion

In this systematic review and meta-analysis, we identified 33 genetic studies of PCV. After the individual results were pooled, the *LOC387715* rs10490924, *HTRA1* rs11200638, *CFH* rs1061170, rs800292, rs3753394, rs1329428 and rs1410996, and *C2* rs547154 were found to be significantly associated with PCV. In addition, by comparing the variants between PCV and AMD, only the rs10490924 showed a statistical difference. Furthermore, by analyzing the genotype-phenotype correlation, we found that the risk genotypes of rs10490924 were associated with larger lesion size, greater chance of vitreous hemorrhage, and worse response to therapy in PCV.

To date, a group of genetic risk factors for AMD has been identified. This helps us understand not only the etiology and pathogenesis but also the diagnosis and management of this ophthalmic condition. In view of the phenotypic similarities between AMD and PCV, the genes for AMD are good candidates for genetic studies of PCV. Until now, almost all reported AMD-associated genes have been investigated in PCV, including *LOC387715*, *HTRA1*, *CFH*, *C2*, *CFB*, *C3*, *SERPING1*, *PEDF*, *Elastin*, *TLR3*, *RDBP*, and *SKIV2L*.

With this systematic review and meta-analysis, the association of *LOC387715* rs10490924 was confirmed, with the overall allelic OR 2.27. The homozygote and heterozygote ORs were 4.90 and 1.74, respectively, suggesting an additive genetic effect. A study reported that *LOC387715* encoded a mitochondrial membrane protein and was expressed in the retina [46]. The association of *LOC387715* with PCV suggests that mitochondrial disorders may play an important role in PCV pathogenesis. Another variant at the 10q26 region, *HTRA1* rs11200638, was also confirmed to be associated with PCV. A study reported that *HTRA1* transgenic mice had retinal pigment epithelium induced choroidal branching vascular networks, polypoidal lesions, severe degeneration of the elastic laminae, and tunica media of choroidal vessels [47], suggesting that overexpression of HTRA1 may predispose individuals to PCV. To date, whether the *LOC387715* or the *HTRA1* at 10q26 is the gene responsible for AMD and PCV remains in question because of the strong linkage disequilibrium between them. However, this issue cannot be solved in our current meta-analysis, and awaits further functional characterizations. The associations of *CFH* rs1061170, rs800292, rs3753394, rs1329428, and rs1410996, and *C2* rs547154 were also confirmed, suggesting that the complement system and inflammatory pathways may also play an important role in the pathogenesis of PCV. The variants *RDBP* rs3880457, *SKIV2L* rs2075702, and *C3* rs2241394 were also reported in one article to be associated with PCV, suggesting a role for the immunological system in PCV. In contrast, polymorphisms at *SERPING1*, elastin, *SOD2*, *PEDF*, and *TLR3* were not associated with PCV.

There are some common and distinct clinical characteristics between PCV and AMD. Both PCV and wet AMD usually involve older adults. However, patients with PCV tend to be younger. The prevalence of AMD is higher in Caucasians than in Asian and black populations, while the prevalence of PCV is higher in Asians and Africans than in Caucasians. Eyes with PCV usually lack drusen—a characteristic sign of early AMD. However, some cases have demonstrated clinical manifestations of PCV and dry or wet AMD. Although PDT and anti-vascular endothelial growth factor (VEGF) therapies are therapeutic opinions for PCV and AMD, the responses to treatments between these two diseases are different. PCV seemingly has a better response to PDT but poorer response to anti-VEGF agents such as bevacizumab [48]. In view of such controversies, whether PCV is a subtype of AMD or a specific entity of disorder remains unsolved, and one solution is to compare the genetic etiology of PCV and AMD.

Through a systematic review, we identified 22 articles reporting the genetic associations of 11 genes with PCV and AMD, including *LOC387715*, *HTRA1*, *CFH*, *CFB*, *C2*, *SERPING1*, elastin, *SOD2*, *PEDF*, *TLR3*, and *9p21*. Among them, only *LOC387715* rs10490924 was statistically different between PCV and AMD, with an allelic OR of 0.66 (95% CI: 0.57–0.76, p<0.00001). This difference suggests that although *LOC387715* is associated with PCV and AMD, its effect could be less strong in PCV than in AMD. In view of the distinct difference in the prevalence of PCV between Caucasian and Asian populations with a comparable frequency of the risk allele, there could be yet-to-be-identified genetic or environmental factors guiding the development of each phenotype. However, the variants in other genes were not statistically different between PCV and AMD, including *HTRA1* rs11200638, *CFH* rs1061170, *CFH* rs800292, and *C2* rs547154. The failure in differentiation may be due to the small overall ample size from a limited number of studies, especially for the eight variants studied in only one article, and needs further investigation.

Genotype-phenotype correlation may shed light on the pathogenesis and clinical management of disease. In this meta-analysis, we found that *LOC387715* rs10490924 was statistically associated with lesion size and vitreous hemorrhage in PCV, with the risk genotype TT associated with a larger lesion and a greater risk of vitreous hemorrhage, that is, more severe phenotypes. This may support the role of *LOC387715* in the pathogenesis of PCV. The association of rs10490924 with gender, age of onset, bilaterality, and BCVA is controversial. The association of *HTRA1* rs11200638 and bilaterality or BCVA is also controversial. The rs10490924 genotype was also correlated with the therapeutic response in PCV. The risk genotypes, TT or TG, are associated with poorer therapeutic response, and the mean difference was 0.39 and 0.20 LogMAR lines, respectively. These results provide pharmacogenetics evidence for estimating the visual prognosis after therapies for PCV.

One advantage of this systematic review and meta-analysis is an overview of all published genetic studies in PCV, demonstrating the overall effects and, at least partially, resolving the controversies. There are also some limitations in this study. First, the number of original studies was limited for some genes, and the conclusions may not be sufficiently strong. Second, the quality of the meta-analysis depends on the quality of the original studies. In some studies, the HWE was not examined in the control group; thus, quality control was lacking. Third, there was significant heterogeneity among studies of some polymorphisms. The source of heterogeneity may include the small sample size in some studies and the different clinical characteristics of patients in different studies. A random effect model was used for the meta-analysis when statistically significant heterogeneity was met. Since most reports were by Eastern Asians, especially Japanese, and there was only one report by one group about a Caucasian population, no subgroup analysis was performed. Fourth, there may be an imbalance between the case and control groups. For example, the gender or age may be different between groups in some articles. The imbalance can be corrected with multivariate analysis in the original studies, but cannot be handled in the meta-analysis. This may also be a cause of heterogeneity. Fifth, we did not search Japanese databases. Although some Japanese medical journals are indexed in PubMed and Embase, we still may have missed some articles in Japanese or other languages. Finally, only one GWAS of PCV [29] has been published, and we could not perform a meta-analysis of GWASs to identify new loci/polymorphisms associated with PCV.

In conclusion, in this systematic review and meta-analysis of 33 articles reporting genetic associations in PCV, polymorphisms at *LOC387715*, *HTRA1*, *CFH*, and *C2* were found to be significantly associated with PCV. *LOC387715* rs10490924 was the only variant showing a significant difference between PCV and AMD. This variant was also correlated with lesion size, vitreous hemorrhage, and therapeutic response in PCV. Further investigations are necessary to confirm the roles of those genes reported in a limited number of original studies.

## **Appendix 1**. Meta-analysis of the association of *LOC387715* rs10490924 with polypoidal choroidal vasculopathy in different genetic models.

Squares indicate study-specific OR; the size of the box is proportional to the weight of the study; horizontal lines indicate 95% confidence interval (CI); diamond indicates summary OR with its corresponding 95% CI. **A**: homozygote; **B**: heterozygote; **C**: dominant model; **D**: recessive model; **E**: additive model. To access the data, click or select the words “Appendix 1.” This will initiate the download of a compressed (pdf) archive that contains the file.

## **Appendix 2**. Meta-analysis of the association of *HTRA1* rs11200638 with polypoidal choroidal vasculopathy in different genetic models.

Squares indicate study-specific OR; the size of the box is proportional to the weight of the study; horizontal lines indicate 95% confidence interval (CI); diamond indicates summary OR with its corresponding 95% CI. **A**: homozygote; **B**: heterozygote; **C**: dominant model; **D**: recessive model; **E**: additive model. To access the data, click or select the words “Appendix 2.” This will initiate the download of a compressed (pdf) archive that contains the file.

## **Appendix 3**. Meta-analysis of the association of *Complement factor H* rs1061170 with polypoidal choroidal vasculopathy in different genetic models.

Squares indicate study-specific OR; the size of the box is proportional to the weight of the study; horizontal lines indicate 95% confidence interval (CI); diamond indicates summary OR with its corresponding 95% CI. **A**: homozygote; **B**: heterozygote; **C**: dominant model; **D**: recessive model; **E**: additive model. To access the data, click or select the words “Appendix 3.” This will initiate the download of a compressed (pdf) archive that contains the file.

## **Appendix 4**. Meta-analysis of the association of *Complement factor H* rs800292 with polypoidal choroidal vasculopathy in different genetic models.

Squares indicate study-specific OR; the size of the box is proportional to the weight of the study; horizontal lines indicate 95% confidence interval (CI); diamond indicates summary OR with its corresponding 95% CI. **A**: homozygote; **B**: heterozygote; **C**: dominant model; **D**: recessive model; **E**: additive model. To access the data, click or select the words “Appendix 4.” This will initiate the download of a compressed (pdf) archive that contains the file.
